# Pathologic Fractures: A Neglected Clinical Feature of Parathyroid Adenoma

**DOI:** 10.1155/2010/357029

**Published:** 2010-11-29

**Authors:** Hassan Abshirini, Iran Rashidi, Nader Saki

**Affiliations:** ^1^Otolaryngology, Head and Neck Surgery, Imam Khomeini Hospital, Ahvaz Jundishapur University of Medical Sciences, P.O. Box 61537-15794, Ahvaz, Iran; ^2^Pathology, Ahvaz Pathologic Department, Golestan Hospital, Ahvaz, Iran

## Abstract

The pattern of clinical presentation of primary hyperparathyroidism (pHPT) has changed dramatically from a severe disease to an asymptomatic condition in Western countries. The story is completely different in Eastern countries. Bone and joint related sign and symptoms like bone pain and multiple fractures are common in these patients. Imaging and nuclear medicine studies will be helpful specially in patient who candidate for surgical removal of the abnormal parathyroid gland. Here, we present a 48-year-old man with multiple typical fractures in long bones and a single adenoma in his right inferior parathyroid gland. pHPT is a severe, symptomatic disease with serious complications and high morbidity in Iran. Advanced skeletal disease is the most common pattern of presentation.

## 1. Introduction

The main effect of parathyroid hormone (PTH) is to increase the concentration of plasma calcium level by increasing the release of calcium and phosphate from the bone matrix, increasing calcium reabsorption by the kidney and increasing renal production of 1.25 dihydroxy vitamin D-3 (Calcitriol) which elevate level of plasma calcium [1, 2]. PTH also causes phosphaturia, thereby decreasing serum phosphate level [1, 3, 4].

Usually, four parathyroid glands are located posterior to the thyroid gland. Primary hyperparathyroidism (pHPT) is a disease characterized by hypercalcemia attributable to autonomous overproduction of PTH. Although some patients with pHPT may have normal serum calcium concentrations, but most of them have hypercalcemia. Therefore, pHPT can often be detected by routine serum calcium measurement. pHPT is present in about 1% of the adult population. The incidence of the disease increases to 2% or higher after age 55 and is 2 to 3 times more common in women than in men [5, 6]. In approximately 80–85% of cases with primary hyperparathyroidism, a single adenoma is found [2, 5]. Multiple gland hyperplasia or neoplasia is present in the 15% remaining [4, 7, 8]. pHPT is the commonest cause, affecting approximately 4 per 100,000 populations per annum and has a peak age of incidence of 50–60. It affects females more than males with a ratio of 3 : 1 [2, 4]. pHPT is a severe, symptomatic disease with serious complications and high morbidity in Iran. Advanced skeletal disease is the most common pattern of presentation at a young age [2].

## 2. Case Report

**Figure 1:**
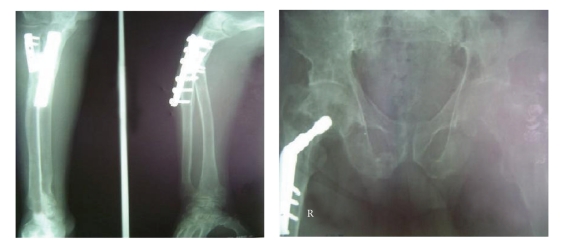
The fractures that were treated by open reduction and internal fixation.

A 48-year-old man was referred by an orthopedic surgeon due to suspicious generalized osteoporosis and multiple long bone fractures. He had two long bone fractures in his right extreme upper and lower points occurred during last six months, with negative history of the major traumatic events. The patient fractures were managed by open reduction and internal fixation surgery, respectively (Figure 1). On the first visit, he was complaining from generalized bone pain and muscle weakness. He also showed a history of polyuria and polydipsia with a clear history of severe renal colic with the passage of large *stones*. Laboratory data obtained are summarized in Table 1. 

**Figure 2:**
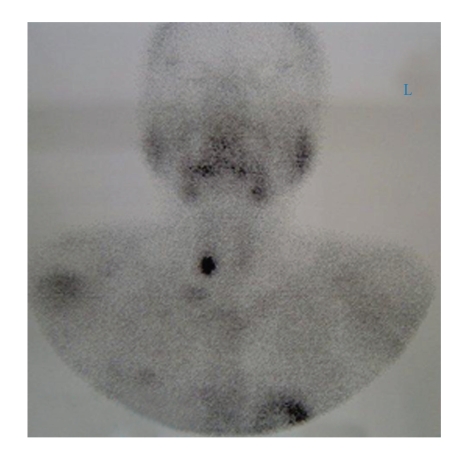
The T99m nuclear scan a focal and persistent active spot at the lower pole of the right thyroid gland, consistent with parathyroid adenoma.

Ultrasonographic examination of kidneys and thyroid gland revealed multiple kidney stones in both side and a well-defined hypoechoic mass measuring 12 × 13 × 11 mm in the right inferior thyroid lobe. A generalized osteoporotic feature was obvious in extremities, thoracic and lumbosacral vertebrae, and iliac bones. At the T99m MIBI nuclear scan a focal and persistent active spot at the lower pole of the right thyroid gland, consistent with parathyroid adenoma was detected (Figure 2). A classic neck exploration with a horizontal thyroid incision was performed. A 13 × 14 mm yellowish brown rubbery mass at the inferior parathyroid region on the right side was detected and excised. Histological study confirmed the diagnosis of parathyroid adenoma. All other three glands were examined grossly during surgery and no abnormality was found. Serum calcium level returned to normal level (9.7 mg/dl) in 24-hour postoperatively. PTH level on 3rd postoperative day was in the normal range. Patient complaint of muscle weakness and bone pain disappeared during the first week postoperatively.

## 3. Discussion

There are striking similarities between clinical and laboratory findings of pHPT from Iran and other eastern regions [8, 9]. Testing of intact PTH level is the core of diagnosis of hyperparathyroidism [3]. Elevated PTH and serum-ionized calcium levels is a diagnostic method for pHPT [4]. A 24 hour urinary calcium measurement is necessary to rule out familial hypocalciuric hypercalcemia [4]. Patients with pHPT usually excrete more than 200 mg calcium per 24-hour, a calcium/creatinine clearance ratio <0.01 [4]. Imaging studies are not used to diagnosis, confirm, and to decide on surgical therapy of hyperparathyroidism [9, 10]. If a limited parathyroid exploration is to be attempted, a localizing study is necessary [9–11]. In patients who have recurrent or persistent hyperparathyroidism after a previous surgery, an imaging study will be necessary [12], in which the T99m nuclear scan is the best initial test [4, 10]. T99m nuclear scan is highly specific for abnormal parathyroid tissue, and its sensitivity is more than 90% in solitary adenoma, but in multiglandular disease its sensitivity is very low (55%) [10].

Combination of ultrasound Computed Tomography (CT) has incremental value in accurately localizing solitary parathyroid adenomas over either technique alone [10]. Ultrasonography, CT scans, and Magnetic Resonance Imaging (MRI) all have been used for localization, but they have been replaced largely with 99mTc-sestamibi.

In the case of recurrent or persistent disease and in ectopic locations such as the mediastinum particularly, MRI may be useful [4, 9, 10]. Bilateral internal jugular venous sampling for parathyroid hormone determination may be used in patients with nonlocalized pHPT. Subperiosteal bone resorption and osteitis fibrosa cystica now are less commonly seen in pHPT. Osteitis fibrosa cystica (brown tumor) was seen only in 10–15% in older reports but new is seen rarely, because of increased incidence of milder forms of disease [7]. The pathognomonic feature of disease increased giant multinucleated osteoclasts in scalloped areas of the surface of the bone (How ship's lacunae) and replacement of the normal cellular and morrow element, by fibrous tissue [2]. Suspecting malignancy, the clinician should be highly alert to other possible causes of bony lesions. Brown tumor should be kept in mind during our practice [13]. Multiple maxillofacial brown tumors can be the primary hyperparathyroidism manifestation [14]. In this case decreased bone density and two pathologic fractures in the neck of the right femur and in the radius and ulna of the right hand were obvious on the first visit. We should remember that several types of malignancies present in the lung, head and neck, esophagus, breast, and renal cells, can cause paraneoplastic hypercalcemia and mimic signs and symptoms of parathyroid adenoma [15]. In the last 30 years the most contemporary series show an incidence of 20% or less. In most published series of patients presenting with urolithiasis the incidence of concurrent PHPT is between 2% and 8% [16]. Greater than 50% of patients with hyperparathyroidism have renal symptoms manifested by nephrolithiasis and nephrocalcinosis [17]. Recurrent acute pancreatitis can be the first and sole presentation of undiagnosed pHPT. Muscle weakness, particularly in the proximal extremity muscles, together with progressive fatigue and malaise, may occur in symptomatic pHPT [4]. Various degrees of depression, nervousness, and cognitive dysfunction may commonly occur in pHPT [4].

Hypertension is more prevalent among patients with hyperparathyroidism [18]. Nonspecific renal, neuralgic, gastrointestinal, or bone and muscle system signs and symptoms, can mislead the physician and cause significant delay in diagnosis. As you see in the present case, he had complained from those symptoms for more than 18 months and two previous surgeries without definite diagnosis during last six months.

Some clinicians advocate surgical therapy in all patients with primary hyperparathyroidism but currently acceptable indications for surgery, if serum calcium level is less than 11.5 mg/dl, symptomatic disease presents, and 24-hour urinary calcium excretion more than 400 mg [4–6]. Our patient satisfied all of these criteria. The standard operation is complete neck exploration with identification of all parathyroid glands and removal of all abnormal glands. In the case of 4 gland disease, 3.5 gland parathyroidectomy must be performed. Approximately 50–70 mg of the most normal appearing glands must be left intact [4, 8, 11]. In this case three other parathyroid glands examined during surgery, all was normal thus only the adenomatous gland was removed. Parathyroidectomy reduced the risk of fracture in all patients with pHPT, when compared with observation [19, 20]. The benefits of parathyroidectomy were reported in all patients with pHPT, regardless of age, calcium level, or bone mineral density [20]. Although, offering parathyroidectomy to all patients with pHPT, regardless of age and other variables may have good advantages, but it should be considered very carefully in older patient [21]. A recent cost analysis study emphasizes the importance of early parathyroidectomy, demonstrating that parathyroidectomy is cost-saving compared to observation and serial monitoring of patients with PHPT.

## Figures and Tables

**Table 1:** Laboratory data of patient with their related normal range.

Laboratory variables	Patient's values	Normal values
Calcium, mg/dl	11.7	8.6–10.6
Phosphor, mg/dl	2	2.5–5
Alkaline phosphate, u/l	2721	100–290
24-hour urine calcium, mg	700	100–300
Parathyroid hormone, pg/mL	190	9–55
